# Diagnostic Evaluation of Automatic Noncontact Ultra-Wideband Radar Combined With Oximeter for Obstructive Sleep Apnea in the South of China

**DOI:** 10.1155/carj/6631384

**Published:** 2025-09-03

**Authors:** Shuyue Wang, Jian Wu, Haiyao Zheng, Yan Ruan, Feng Yu, Tao Liao

**Affiliations:** ^1^Department of Otorhinolaryngology-Head and Neck Surgery, Guangzhou Red Cross Hospital (Guangzhou Red Cross Hospital of Jinan University), Guangzhou 510220, China; ^2^Department of Otolaryngology Head and Neck Surgery, The First Affiliated Hospital of Guangzhou University of Chinese Medicine, Guangzhou 510405, China; ^3^Institute of Otolaryngology Head and Neck Surgery, Jinan University, Guangzhou 510220, China; ^4^Lingnan Institute of Otolaryngology, Guangdong Clinical Research Academy of Chinese Medicine, Guangzhou 510405, China

**Keywords:** diagnosis, obstructive sleep apnea, ultra-wideband radar

## Abstract

**Background:** Polysomnography (PSG) is the traditional technique for diagnosing obstructive sleep apnea (OSA) with some limitations. Ultra-wideband radar (UWB) is a new method for diagnosing OSA that combines multiple techniques including radar technology, artificial intelligence (AI), and big data algorithms. The accuracy of UWB in OSA diagnosis needs further scientific verification, especially in southern China.

**Methods:** Fifty patients from southern China wore UWB with oximetry and PSG simultaneously overnight. UWB generated automated reports; PSG was manually interpreted. Lowest oxygen saturation (LSpO2) and apnea-hypopnea index (AHI) from both methods were compared.

**Results:** High correlation was found between UWB and PSG for AHI (*r* = 0.925, *p* < 0.001) and LSpO2 (*r* = 0.990, *p* < 0.001). The average bias of AHI (−3.09, *p*=0.98, 95% CI −15.48 − 9.31) and LSpO2 (−0.34, *p* > 0.99, 95% CI −3.68 − 3.04) between the two methods was small. ROC analysis showed good diagnostic performance of UWB versus PSG (AUC = 0.979, *p* < 0.001), with 95.8% sensitivity and 100% specificity.

**Conclusion:** The study demonstrated UWB combined with oximetry could be a reliable alternative to PSG for diagnosing OSA in the south of China.

## 1. Introduction

Sleep apnea syndrome (SAS) is a serious common disease caused by sleep-disordered breathing that endangers vital organs of human beings. Since the research of SAS started by the American medical community in the 1970s, it has garnered increasing attention and rapid development from various professional physicians worldwide. The etiology can be divided into three types: obstruction, centrality, and mixture [1]. Obstructive SAS is the most prevalent in clinical practice, accounting for approximately 50%–70% of cases. Obstructive sleep apnea (OSA) occurs when the upper airway is partially or completely obstructed due to structural abnormalities and/or dysfunction, leading to apnea and/or hypoventilation [2]. This can cause severe complications in the cardiovascular and cerebrovascular systems, central nervous system, endocrine system, and other vital organs [3]. The diagnostic criteria for OSA were the episodes of apnea/hypopnea ≥ 30 times during 7 h of nighttime sleep or a sleep apnea-hypopnea index (AHI) ≥ 5 [4].

Polysomnography (PSG) is the traditional gold standard for diagnosing OSA, but it has some drawbacks [4]. It requires complicated operation by specially skilled personnel. Multiple electrodes have to be attached to the patient, which can be uncomfortable and disrupt sleep; what's more, electrode displacement during sleep may compromise the monitoring results. Hence, the first-night effect of PSG often prevents accurate assessment of the patient's true sleep status [5]. Children often cannot tolerate the discomfort during the PSG monitoring. The high cost of PSG also limits its accessibility. Therefore, a more comfortable, convenient, and accurate alternative method is urgently needed.

Ultra-wideband radar (UWB) introduced by our hospital has several innovative features, which is a useful medical application combining multiple techniques including radar technology, artificial intelligence (AI), and big data algorithms. This device can detect the patient's breathing conditions during sleep using radar waves which can penetrate nonmetallic media such as clothes and bedding without direct contact with the patient [6]. The sleep breathing status was monitored throughout full-night time by UWB; meanwhile the oxygen saturation and heart rate were monitored by a medical ring-shaped oximeter combined with the major UWB device. All the detected data can be automatically analyzed by the software provided by the device, and the existence and severity of OSA can be judged by AI. Unlike traditional PSG or portable contact monitors, UWB does not require direct contact with the patient, eliminating the discomfort caused by electrodes, wires, sensors [7], etc. This device provides a representation of the patient's sleep breathing condition, entirely without influence of the first-night effect.

In southern China, the hot and humid climate poses unique challenges for sleep monitoring. Persistent sweating in this region frequently compromises the adhesion and accuracy of traditional PSG skin electrodes, leading to frequent signal interference or even monitoring failures. This issue is particularly pronounced in OSA suspects, where motion-induced electrode detachment during sleep further undermines diagnostic reliability. Meanwhile, as the most densely populated regions in China, southern China faces a disproportionately high burden of OSA with a large requirement for sleep apnea screening. The first-night effect of PSG often necessitates repeat testing for patients, exacerbating the strain on limited diagnostic resources and urgent patient demand in southern China's densely populated regions. This bottleneck highlights the critical need for alternative solutions like noncontact UWB to alleviate pressure on healthcare systems in the south of China. While evidence for UWB accuracy is still insufficient at present, though two recent studies on UWB have been conducted in northern China [8, 9], evidence from southern China is lacking. Regional variations in factors like living environment and physiology may impact OSA presentation and diagnosis. Research on geographically diverse populations is valuable to support UWB applications. The UWB technology investigated in this study is currently in the clinical promotion stage within China and has not yet gained widespread acceptance in the international sleep medicine community. Thus, to date, no research institutions outside China have initiated related studies. In contrast, recent advancements in Western countries predominantly focus on evaluating whether contact-dependent wearable devices, such as smart ring oximeters, can replace traditional PSG. Notably, such devices rely on direct skin contact and optical sensors, which represent a distinct technological pathway compared to the noncontact UWB explored here.

This study aims to verify the diagnostic accuracy of UWB compared to PSG in southern China. In this research 50 patients with suspected OSA were monitored simultaneously using both PSG and UWB combined with an oximeter from May to September 2019; the monitoring results obtained from the two methods were meticulously compared and analyzed.

## 2. Methods

### 2.1. Features of Patients

From May to September 2019, 50 snoring patients who were admitted to the outpatient clinic of our hospital were enrolled in this study. All patients were from Guangzhou, the largest city in southern China, including 36 males and 14 females, with ages ranging from 28 to 75 years. Before monitoring, routine physical examinations were conducted for every patient, and some data was collected including height, weight, body mass index (BMI), blood pressure, and heart rate (Table 1). The examination of maxillofacial morphology, nasal cavity, oral cavity, pharynx, larynx, and cardiopulmonary function was also performed. We ensured full communication with all patients involved in this research and obtained their informed consent.

### 2.2. Monitoring Method

All patients underwent monitoring in a dedicated sleep monitoring room for a minimum of 7 h one night, using both UWB combined with an oximeter and PSG simultaneously. The PSG data for each patient were analyzed and interpreted by the same clinician. The UWB device automatically interpreted the monitoring data and reported the results, which were then manually reviewed before the final report was issued.

PSG captures seven main parameters including electroencephalogram (EEG), electro-oculogram (EOG), submental and leg electromyogram (EMG), electrocardiogram (ECG), chest and abdominal respiratory movement, oronasal air-flow, oxygen saturation, and periodic leg movement in sleep.

The UWB detects parameters including chest and abdominal respiratory movement, oronasal air-flow, oxygen saturation, and heart rate. Subsequently, monitoring data were analyzed by AI to identify events such as sleep apnea and hypopnea, and the software automatically recorded sleep events and manually calculated the AHI.

## 3. Equipment

**Figure 1:**
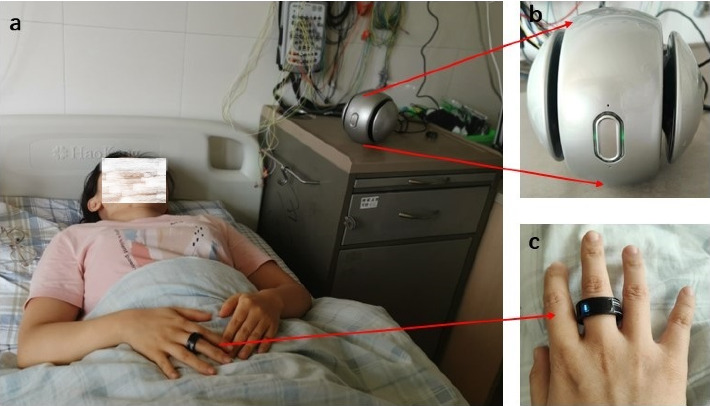
The method of wearing and using UWB. (a) Monitoring with UWB. (b) The lightweight radar ball. (c) The medical ring‐shaped pulse oximeter.

The UWB device consists of an UWB monitor whose appearance is a lightweight ball, a medical ring-shaped pulse oximeter, and an Android iPad analyzer (device model number: ZG-S01B, provided by Zhao Guan Technology, China). The spherical radar monitor was placed on bedside table within 1 m from the patient's chest and 10 patients above the patient's mattress, and the antenna of the radar was set toward patient's thoracic region. The pulse oximeter is designed to look like a ring which comes in four sizes to suit different body shapes and children (Figure 1). Data were wirelessly transmitted to the tablet and analyzed using proprietary algorithms in the Verson 1.0 software called Sleep Respiratory Disorder Screening Software, which has been optimized for accurate detection and analysis of sleep apnea-related parameters.

The PSG device with 32 channels consists of three main components: patient unit, communication unit, and bedside unit (N7000, Embla system, USA).

### 3.1. Diagnostic Criteria

In this study, the diagnostic criteria and severity grading standards for OSA in all patients were according to Expert consensus on diagnosis and treatment of OSA and metabolic syndrome (2022) [10]. The criteria for hypopnea is a decrease in oronasal air-flow of ≥ 30% from baseline, accompanied by a decrease in oxygen saturation of ≥ 3% or awakening, and lasting for ≥ 10 s. The criteria for apnea are defined as a decrease in oronasal air-flow of ≥ 90% from baseline, and lasting for ≥ 10 s. Obstructive apnea (OA) refers to the presence of chest and abdominal breathing movement during episodes of hypopnea or apnea. The AHI refers to the sum of the average number of apneas and hypopneas per hour during sleep. OSA was classified as mild (5 ≤ AHI < 15), moderate (15 ≤ AHI < 30), or severe (AHI ≥ 30) based on the AHI. Patients with OSA were classified into three grades according to their lowest oxygen saturation (LSpO2) during sleep: mild hypoxemia (85% ≤ LSpO2 < 90%), moderate hypoxemia (80% ≤ LSpO2 < 85%), or severe hypoxemia (LSpO2 < 80%) (Table 2).

### 3.2. Statistics

The continuous variables from patients were described by $\overset{¯}{X}$ ± *s*. The Pearson correlation test was used to analyze the correlation of the data obtained by UWB and PSG. Bland–Altman plots were used to assess the agreement between the data of UWB and PSG. Receiver operating characteristic (ROC) analysis was used to determine the sensitivity and specificity of UWB against PSG as the reference standard. The area under the ROC curve (AUC) was calculated to summarize the diagnostic accuracy. Statistical analysis of the data was performed using SPSS25 statistical software, and a *p* value < 0.05 was considered statistically significant.

## 4. Results

### 4.1. Diagnosis and Classification of Patients Using PSG and UWB

Out of the 50 total patients, PSG diagnosed 47 with OSA, including five mild cases, 18 moderate cases, and 24 severe cases. The remaining three patients were found to be normal via PSG. In comparison, the UWB method diagnosed 46 patients with OSA, comprising seven mild cases, 16 moderate cases, 23 severe cases, and four patients who were classified as normal.

### 4.2. Correlation and Agreement of AHI and LSpO2 for the Two Monitoring Methods

**Figure 2:**
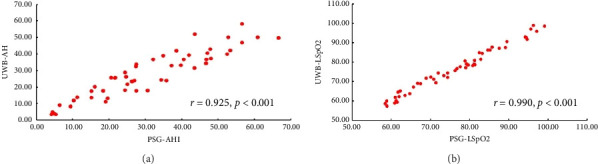
Correlation analyses of AHI and LSpO2 for two methods. (a) Correlation between PSG‐AHI and UWB‐AHI. (b) Correlation between PSG‐LSpO2 and UWB‐LSpO2.

**Figure 3:**
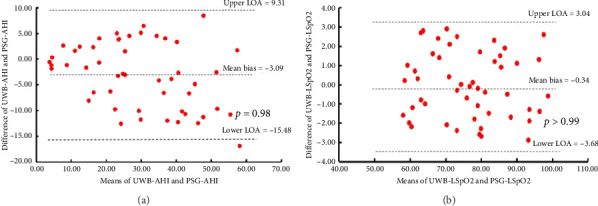
Bland–Altman plots of AHI and LSpO2 for two methods. (a) UWB-AHI/PSG-AHI agreement was estimated according to Bland–Altman comparison. (b) UWB-LSpO2/PSG-LSpO2 agreement was estimated according to Bland–Altman comparison.

AHI and LSpO2 of each patient obtained from PSG and UWB showed normal distribution, and the mean and standard deviation of AHI and LSpO2 are shown in Table 1. Meanwhile, the scatter plots of AHI and LSpO2 obtained from the two methods show a strong degree of similarity. When considering the correlation analysis results, the AHI measured by UWB and PSG displayed a highly positive correlation (*r* = 0.925, *p* < 0.001) (Figure 2(a)), while LSpO2 also exhibited a highly positive correlation (*r* = 0.990, *p* < 0.001) (Figure 2(b)). The Bland–Altman plots revealed significantly high correlation and good agreement between UWB-AHI and PSG-AHI (mean bias −3.09, 95% confidence interval: −15.48 − 9.31, *p*=0.98) while similar results were obtained between UWB-LSpO2 and PSG-LSpO2 (mean bias −0.34, 95% confidence interval: −3.68 − 3.04, *p* > 0.99) (Figure 3). The strong correlation and agreement between UWB and PSG methods demonstrate that UWB monitoring can produce highly comparable AHI and LSpO2 results to the gold standard PSG for OSA diagnosis.

### 4.3. Diagnostic Performance of the UWB Method Assessed by ROC Analysis

**Figure 4:**
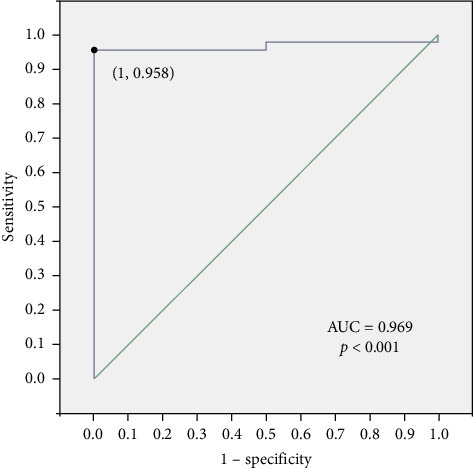
Diagnostic performance of UWB for OSA diagnosis compared to PSG.

ROC analysis was performed to assess the sensitivity and specificity of UWB against PSG as the gold standard. The AUC value was 0.979, indicating good diagnostic performance of UWB for OSA diagnosis compared to PSG. At the optimal cutoff threshold, the UWB device had a sensitivity of 95.8% and specificity of 100% for identifying OSA patients (*p* < 0.0001) (Figure 4). These results demonstrate that the novel UWB monitoring device shows considerable promise as an accurate tool for OSA diagnosis compared to the current gold standard, PSG.

## 5. Discussion

PSG is currently considered the gold standard for diagnosing OSA internationally; however, the process of PSG monitoring has a number of shortcomings [4, 11]. PSG operation is a complicated and time-consuming process that requires multiple electrodes to be in contact with the patient's body for an extended period [12]. This can lead to discomfort and low compliance among patients, and the first-night effect can make it difficult to accurately reflect the patient's sleep status, ultimately affecting the final result [5, 13]. Besides, the PSG equipment is very expensive, with prices starting around US$200,000 for the most affordable model, which most primary hospitals and developing countries cannot afford. This greatly limits the application of PSG. In contrast, the UWB device used in this study only costs around US$20,000, which is much more affordable and provides broader access. Therefore, we explored the possibility of using UWB which was a novel, portable, wireless AI device, as a replacement for PSG in diagnosing OSA and determining its severity [6].

To verify the reliability and accuracy of UWB, it is necessary to first check the consistency of the monitoring results of the two methods under synchronous monitoring conditions. Although the validation of the spheroidal UWB radar for sleep apnea detection was first reported in a study in 2020 by Zhou et al. [7], their research suffered from some flaws. In their study, the UWB device was the first generation, only chest and abdominal movement and respiratory air-flow were detected, and the cardiac variable was deficient. In our study, the second-generation UWB device was applied in combination with a medical ring-shaped oximeter attached by manufacturer to measure cardiac variables including oxygen saturation and heart rate. Both AHI and LSpO2 can be obtained which were more comprehensive for the diagnosis and severity evaluation of OSA associated with hypoxemia [14, 15]. In 2021 and 2022, two recent studies from the same team demonstrated the potential of contact-free UWB monitoring to replace PSG, however their research was geographically limited to Beijing, the most major city in northern China [8, 9]. Further evidence from broader regions of China is needed to validate the universal applicability of these technologies across diverse populations and environments in China. The generalizability and accuracy of contact-free screening requires further verification outside major northern cities. Our study conducted in southern China demonstrates that UWB radar combined with oximetry could serve as a reliable alternative to traditional PSG for diagnosing OSA. The accuracy of UWB was verified against PSG in a southern Chinese population for the first time, which provides an important supplement to previous evidence on UWB application which was geographically limited to northern China.

The monitor method of UWB radar combined with oximeter is classified as Type III sleep test device according to “Clinical Practice Guideline for Diagnostic Testing for Adult OSA: An American Academy of Sleep Medicine Clinical Practice Guideline” [16, 17]. UWB is capable of detecting biomedical signals including respiratory movement and body motion, as well as counting the patient's sleep events and frequency. As it does not require any sensors to be attached to the patient, this portable radar device can work in a more child-friendly noncontact way. Unlike PSG, which uses chest and abdominal belts to sense breathing motion [8], UWB directly captures extremely subtle respiratory movement and body motion using wireless radar technology. The monitoring data are collected by an Android iPad analyzer attached by manufacturer and the sleep reports of patients can be analyzed by AI using bid data and also reviewed manually.

UWB in this study has several significant features. Firstly, contactless monitoring increases patient compliance and comfort and greatly reduces the impact of the first-night effect. For children, during the traditional PSG monitoring process, the attachment of multiple electrodes may cause discomfort and resistance, affecting the accuracy of monitoring results. The noncontact monitoring method of UWB avoids this problem and is more acceptable to children. However, children's sleep patterns and respiratory characteristics are different from those of adults. Further research is needed to determine the monitoring accuracy and diagnostic thresholds of UWB in the pediatric population. For the elderly, UWB offers unique advantages over PSG: its noncontact design eliminates interference from loose skin—a common age-related physiological change that often compromises electrode adhesion in PSG. However, elderly populations frequently present with multiple chronic comorbidities and declining respiratory function, leading to heterogeneous manifestations of sleep-disordered breathing. These complexities necessitate tailored diagnostic criteria and algorithm adjustments for UWB-based monitoring. Future studies should focus on age-specific subgroup analyses to validate and optimize UWB's performance in this demographic, ensuring its clinical reliability amid diverse physiological challenges. Secondly, the novel radar technique is safe for clinical application [18], because the working power of biological radar is generally less than 1 mW, and some are even below 0.1 mW which is harmless to the human body. Additionally, it has high accuracy; the UWB radar can distinguish the difference of the micron level and capture extremely small motion signals [19]. Last, with a cost approximately one-tenth of PSG, UWB significantly lowers financial barriers for adoption in resource-limited settings, such as primary healthcare facilities and households, particularly in underdeveloped regions. For primary medical institutions, the reduced upfront investment alleviates budgetary constraints, enabling widespread deployment for preliminary OSA screening. Early detection through such accessible tools can prevent disease progression, thereby minimizing costly complications and reducing the overall burden on healthcare systems. Operationally, UWB eliminates the need for specialized technicians or continuous supervision due to its automated, user-friendly interface, further cutting labor costs. In home-based monitoring scenarios, patients can perform self-assessments with minimal training, enhancing compliance and enabling frequent, real-time tracking of sleep health. This proactive approach not only improves individualized disease management but also alleviates overcrowding in tertiary hospitals. Collectively, these features position UWB as a highly cost-effective solution with the potential to reshape OSA diagnostics across diverse socioeconomic contexts, optimizing both clinical outcomes and healthcare resource allocation. This study still has some limitations. Due to the COVID-19 pandemic in China, the period of this study is short, and the number of research subjects is limited. The data collected from patients were relatively simple; only AHI and LSpO2 were the center of attention, which were major parameters for the diagnosis of OSA [20]. Our previous findings confirmed that the UWB device could be used as a reliable substitute for the PSG. We are planning to expand the sample size and collect more observations such as the determination of sleep stages which was significant for other types of sleep disorders [14, 21]. Thus, long-term follow-up and multicenter studies are warranted in the future.

## 6. Conclusion

This study conducted in Guangzhou, southern China, selected two crucial indicators, AHI and LSpO2, for evaluating UWB against PSG in diagnosing OSA. The results demonstrated high consistency between UWB and PSG, indicating UWB can be a viable alternative to PSG for OSA diagnosis. Our research provides an important supplement to the current evidence on UWB application which was primarily from northern China, with numerous advantages, UWB shows promising potential for expanded use in monitoring sleep-disordered breathing. Further studies across diverse regions of China are warranted to validate UWB in varied populations and environments.

## Figures and Tables

**Table 1:** Summary of patients' demographics.

Item	Mean ± SD
Gender	36 male/14 female
Age (years)	50.2 ± 13.8
Height (cm)	167.9 ± 8.2
BMI	27.0 ± 4.9
UWB-AHI	27.8 ± 14.2
UWB-LSpO2 (%)	76.4 ± 11.7
PSG-AHI	30.9 ± 16.5
PSG-LSpO2 (%)	76.5 ± 11.9

*Note:* Except for the row filled in gender, others show the mean and standard deviation of the data.

**Table 2:** OSA and hypoxemia grading standards.

	Mild	Moderate	Severe
AHI	5 to 15	15 to 30	> 30
LSpO2 (%)	85–90	80–85	< 80

*Note:* The above severity grading standards of OSA and hypoxemia were according to the expert consensus on diagnosis and treatment of obstructive sleep apnea and metabolic syndrome (2022).

## Data Availability

The data that support the findings of this study are not publicly available due to privacy or ethical restrictions surrounding the clinical patient data, while the data are available on request from the corresponding authors.
